# Assessment of the phytochemical composition and antimicrobial properties of *Tapinanthus bangwensis* leaves hosted by the branches of *Persea americana*

**DOI:** 10.1186/s12906-023-03860-w

**Published:** 2023-02-03

**Authors:** Maxwell Mamfe Sakyiamah, Paa Kwesi Gordon, Peter Bolah, Prince Kyei Baffour, Ebenezer Ehun, Olga Quasie, Doris Kumadoh, Mary-Ann Archer, Susana Oteng Mintah, Alfred Ampomah Appiah

**Affiliations:** 1 Phytochemistry Department, Centre for Plant Medicine Research, Mampong-Akuapem, Ghana; 2 Department of Microbiology, Centre for Plant Medicine Research, Mampong-Akuapem, Ghana; 3 Pharmacology and Toxicology Department, Centre for Plant Medicine Research, Mampong-Akuapem, Ghana; 4 Production Department, Centre for Plant Medicine Research, Mampong-Akuapem, Ghana; 5 Department of Pharmaceutics, School of Pharmacy and Pharmaceutical Sciences, University of Cape Coast, Cape Coast, Ghana

**Keywords:** *Tapinanthus bangwensis*, Phytochemical composition, Antimicrobial properties, Alternative therapy

## Abstract

**Background:**

Medicinal plants represent a valuable source for new effective and safe antimicrobial drugs making them an alternative therapy. Existing antimicrobial agents are costly and mostly associated with possible side effects. The aim of the present study therefore, was to assess the antimicrobial property and phytochemical composition of hydroethanolic extract of *Tapinanthus bangwensis* leaves and its fractions.

**Method:**

*T. bangwensis* leaves (harvested from its host plant, *Persea americana*) was extracted by cold maceration with 70% ethanol and further fractionated with different organic solvents using the solvent partitioning method to obtain the crude extract, petroleum ether, chloroform, ethyl acetate and the resulting aqueous fractions. The phytochemical constituents of the extracts were screened and quantified. Also, the TLC of the extracts were analyzed to serve as a fingerprint. Using the agar diffusion and broth dilution methods, the antimicrobial properties of the extracts were assessed.

**Results:**

The study showed that the hydroethanolic (70%) crude extract of *T. bangwensis* leaves and its fractions contain phenolic compounds, flavonoids, saponins, phytosterols and reducing sugars. The phytoconstituents were well extracted into the ethyl acetate fraction than the other fractions evidenced in the high levels (*p* < 0.0001) of saponins (66.47 ± 1.72% w/w), phenolic compounds (77.75 ± 1.06 mg/100 mg GAE) and flavonoids (44.34 ± 0.06 mg/100 mg QE) contents. From the antimicrobial studies, all the microorganisms tested exhibited varying degrees of susceptibility to the extracts with MIC values between 0.78 to 12.5 mg/mL. The crude extract of *T. bangwensis* leaves, its ethyl acetate and chloroform fractions also exhibited lethal antimicrobial activity with MLC between 6.25 to 50 mg/mL.

**Conclusion:**

The crude extract of *T. bangwensis* leaves and its fractions demonstrated antimicrobial properties against *Escherichia coli*, *Staphylococcus aureus*, *Staphylococcus saprophyticus* and *Candida albicans,* thereby representing a potential source of natural antimicrobial agent. Further study is required to identify and isolate antimicrobial compounds from the plant for the development of the natural bioactive antimicrobial agents.

**Supplementary Information:**

The online version contains supplementary material available at 10.1186/s12906-023-03860-w.

## Background

For many years, efforts to curtail infectious diseases caused by microorganisms have proven difficult worldwide. Although increasing knowledge in microbiology has led to the development of several therapeutic agents (such as sulfonamide, trimethoprim, quinolones and nitrofurans), the high cost and possible side effects associated with these agents limit their use [1]. Furthermore, most of these drugs lose their efficacy over time due to the development of resistant strains [2]. Thus, the search for new effective and safe antimicrobial agents without severe side effects has become a paramount goal of biomedical scientists. Medicinal plants serve as a valuable source of natural products for maintaining human health with little or no reported side effects [3, 4].

*Tapinanthus bangwensis* (Engl. & K Krause) Danser (African Mistletoe), belonging to the family Loranthaceae and a member of mistletoes with the genus *Tapinanthus*, is a semi-parasitic plant that grows on edible or non-edible evergreen and deciduous trees. It is widely distributed in Sudan and the Sahelian regions of West Africa [5, 6]. *T. bangwensis* that grow on edible plants are mostly used for medicinal purposes [7]. The medicinal properties of *T. bangwensis,* like the other members of mistletoes, are host dependent [8]. Some hosts on which *T. bangwensis* may be found as wild species or cultivated include citrus plants, *Persea americana, Psidium guajava, Theobroma cacao* and *Manikara zapota* [8]. Owing to the many folkloric uses, African Mistletoe has been described as an all-purpose herb [9]. The leaves are used by traditional medical practitioners for the treatment and management of conditions such as skin diseases, liver disorders, diabetes, hypertension, asthma, epilepsy and cancer [6, 10]. Previous pharmacological studies have reported the antimicrobial properties of several species of the Loranthaceae family, including species within the genus *Tapinanthus* (*T. dodoneifolius* (DC) Danse and *T. bangwensis*) [11, 12]. Notwithstanding, there is still scanty information on the antimicrobial properties of *T. bangwensis*, especially those growing on hosts other than citrus plants. Furthermore, not much information is readily available on the chemistry of many African mistletoes, *T. bangwensis* inclusive. The aim of this study therefore was to assess the antimicrobial property and phytochemical composition of hydroethanolic extract of *T. bangwensis* leaves and its fractions harvested from *Persea americana* as its host plant.

## Materials and methods

### Plant samples

The leaves of *T. bangwensis* were freshly harvested from its cultivated host plant (*Persea americana*) at Mampong – Akuapem, in the Eastern region of Ghana (5º 55′ 25.6' N, 0º 7′ 58.5 W) according to guidelines for plant collection at the Centre for Plant Medicine Research (CPMR), Mampong-Akuapem, Ghana, licensed by the Ghana Forestry Commission. The leaves were identified and authenticated by Mr. Peter Atta-Adjei at the Plant Development Department of CPMR. Voucher specimens (Voucher specimen number – CPMR 503) were prepared and deposited in the herbarium of CPMR. The leaves were air dried, and milled using an electrically powered engine (Vendido stainless steel hammer mill Motor: 5.5 kw electric panel mobile total height: 1200 mm total length: 1500 mm total width: 780 mm, voltage: 220–240 V). The powdered samples were stored in a moisture free, air-tight container until further use.

### Extraction

The milled samples of *T. bangwensis* leaves (1.0 kg) was cold macerated with 3.0 L of 70% ethanol, for 3 days with intermittent stirring using protocol described by Jones and Kinghorn with modifications [13]. The extract was filtered and re-extracted with another 3.0 L of 70% ethanol, for 3 days aimed at achieving exhaustive extraction. The filtrates were pulled together (5.9 L) and evaporated using a rotary evaporator (SB-1200), resulting in a volume of 1.0 L. A portion of the resulting concentrate (approx. 100 mL) was oven dried (50 ºC) to obtain dried extract of *T. bangwensis* leaves (8.064 g) for further analysis.

#### Partitioning of 70% ethanolic crude extract of *T. bangwensis*

The concentrated crude extract of *Tapinanthus bangwensis* leaves (500 mL) was successively partitioned with petroleum ether, chloroform and ethyl acetate. The organic fractions were evaporated to dryness using rotary evaporator (SB-1200) to obtain the petroleum ether fraction (1.845 g), chloroform fraction (2.279 g), ethyl acetate fraction (7.298 g), and the remaining aqueous fraction was oven dried at 50 ºC (9.20 g).

### Phytochemical analysis of extracts

Phytochemical analysis of the crude extract of *T. bangwensis* leaves and its fractions (petroleum ether, chloroform, ethyl acetate and aqueous fractions) was carried out first qualitatively to identify the phytochemical constituents present, and further quantified using established protocols. Total saponins, total phenolic compounds, total flavonoids and total reducing sugar detected in the extracts were quantified as described below;

a **Total saponins**
  The total saponin content of the crude extract and its fractions was determined according to the method described by Roghini and Vijayalakshmi [14]. Briefly, one hundred milliliters (100 mL) of 20% ethanol was added to 1.0 g of the sample (crude extract/fraction). The suspension was then heated with continuous stirring over a water bath (55 °C) for 4 h. The mixture was then filtered and the residue re-extracted with another 200 mL of 20% ethanol. The combined filtrate was reduced to 40 mL over water bath at about 90 °C. The concentrated extract was then transferred into a 250 mL separating funnel and 20 mL of diethyl ether was added. The mixture was shaken vigorously. The aqueous layer was recovered while the ether layer was discarded. To the recovered aqueous layer was added 30 mL of n-butanol repeatedly and washed twice with 10 mL of 5% aqueous sodium chloride. The resultant solution was then evaporated over a boiling water bath, dried in an oven and then weighed. The resultant dry mass was expressed as a percentage of the mass of the starting plant material.
b **Total Phenolic compounds**
  The total phenolic content (TPC) of the crude extract and its fractions was determined using the Folin-Ciocalteu colorimetric method as described by Maria et al. with some modifications [15]. To 25 µL of sample (2 mg/mL) in each well of a 96-well microtiter plate was added 125 µL of Folin-Ciocalteu’s phenol reagent and incubated for 8 min at room temperature (25–28 °C). To the mixture in each well was added 100 µL of saturated sodium carbonate solution (7.5% w/v in water) and mixed gently. The mixture was then kept in the dark for 90 min at 23 °C, after which the absorbance was read at 765 nm using Synergy HTX microplate reader against the reagent blank. The TPC was determined by extrapolating from a calibration curve prepared using gallic acid solution as the standard and expressed as milligrams of gallic acid equivalents (GAE) per 100 mg of dried sample. The estimation of the phenolic compounds was carried out in triplicate.
c **Total flavonoids**
  The total flavonoid content was measured by a colorimetric assay as described by Bibi et al. with some modifications [16]. To 50 µL of each sample (2 mg/mL) in each well of a 96-well microtiter plate was added 15 µL of 5% sodium nitrite. After 5 min of incubation at room temperature, 15 µL of 10% aluminum chloride was added to each well. In 6 min, 100 µL of 1 M sodium hydroxide was added to the mixture. The absorbance was read at 510 nm against a blank. Quercetin was used as standard for the calibration curve. Total flavonoid content of the extract was expressed as mg quercetin equivalents per 100 mg of dried sample (mg/100 mg). All determinations were carried out in triplicate.>
d **Total Reducing sugar**
  Total reducing sugar was quantified based on the Benedict test as described by Hernańdez-Loṕez et al. with modifications [17]. Firstly, the optimal concentration of CuSO_4_ for the Benedict broth was determined by preparing Benedict broth (943 mM sodium carbonate and 670 mM sodium citrate) with different concentrations of CuSO_4_ (27, 54, 108, 135, 162, 217 and 244 mM). The mixtures were heated at 90 ℃ for 5 min on the water bath, cooled and diluted with distilled water (1:5 dilution). The absorbance of the mixture was read at 740 nm on a Microplate reader (Bio Tek Instruments). A standard concentration/absorbance of CuSO_4_ was drawn to determine optimum concentration of CuSO_4_.

Glucose stock solution (0.1%) was mixed with 1 mL of Benedict’s reagent (943 mM sodium carbonate, 670 mM sodium citrate, 217 mM CuSO_4_) and made up to a total volume of 1.5 mL with final concentrations of glucose at 0.3125, 0.625, 1.25, 2.5, 5, 15, 20, 25, and 30 mg mL^−1^. The reaction mixture was heated at 90 ℃ on a water bath for 5 min, cooled and centrifuged for 2 min at 4000 × g. The supernatant was recovered and diluted with distilled water(1:10 dilution). The absorbance was then read at 740 nm to prepare a calibration curve of reacted glucose against absorbance. Analyses were made in triplicate for each concentration.

To 0.5 mL of the extract (30 mg/mL) was added 1 mL of the Benedict reagent as above. The reaction mixture was heated at 90 ℃ on a water bath for 5 min, cooled and centrifuged for 2 min at 4000 × g. The supernatant was recovered and diluted to 1:10 with distilled water. The absorbance was then read at 740 nm to extrapolate the glucose concentration from the calibration curve. Analyses were made in triplicate for each concentration.

### Thin layer chromatography

Two milligram each of the crude hydroethanolic extract and fractions of *T. bangwensis* was dissolved in a suitable solvent. The mixtures were subjected to Thin Layer Chromatography (TLC) as per conventional one-dimensional ascending method using silica gel (Silica gel 60 F_254_). Each sample (1 µl) was spotted on the TLC plate using glass capillaries at a distance of 1 cm. The solvent system, chloroform: methanol (4:1) and dichloromethane: ethyl acetate: methanol (6:2:1) were used to run the plates. The bands were detected by staining the TLC plates with 10% Sulphuric acid and heating over a hot plate (C-MAG HS 10) at 100 °C to develop the chromatograms. The movement of bands was expressed by its retention factor (R_f_) [18].

### Assessment of antimicrobial activity

#### Test organisms

The antimicrobial activity was assessed using two Gram-positive *(Staphylococcus aureus*; *Staphylococcus saprophyticus*) and two Gram-negative bacteria (*Salmonella typhi*; *Escherichia coli*), and two fungi (yeast – *Candida albicans* and mould – *Aspergillus niger*). Pure cultures of *S. aureus* (ATCC 25,923), *S. saprophyticus* (ATCC 15,305), *S. typhi* (ATCC 19,430), *E. coli* (ATCC 25,922), *C. albicans* (ATCC 10,231) and *A. niger* (ATCC 16,888) were obtained from the Department of Microbiology of Centre for Plant Medicine Research in Ghana for the study.

#### Inoculum preparation

Hundred microliters (100 mL) of each stock culture were added to labelled tubes containing 5 mL of Nutrient Broth (Oxoid, UK). The bacteria cultures and *C. albicans,* were incubated at 32.5 ± 2.5 °C for 24 h whiles *A. niger* was incubated at 22.5 ± 2.5 °C for 48 h. A loopful of each tube containing the bacteria/fungi were then streaked unto sterile plates of nutrient agar and malt extract agar (Oxoid, UK), respectively. The bacteria and *C. albicans* inoculated plates were incubated at 32.5 ± 2.5 °C for 24 h while *A. niger* was incubated at 22.5 ± 2.5 °C for 48 h. Colonies of the bacteria and the yeast were introduced into tubes containing 5 mL of nutrient broth using a sterile inoculating loop and incubated at 32.5 ± 2.5 °C (bacteria and *C. albicans*) or 22.5 ± 2.5 °C (*A. niger*) for 3 h. The inoculums were adjusted to 10^8^ CFU/mL and 10^8^ spores/mL before use.

#### Preparation of stock concentrations

Stock solutions of 200 mg/mL were prepared by dissolving 1 g of each extract/fraction (70% ethanolic crude extract, petroleum ether, chloroform, ethyl acetate and aqueous fractions of *T. bangwensis*) in 5 mL of sterile distilled water. The reference standard used against *S. aureus, S. saprophyticus, E. coli* and *S. typhi* was Ciprofloxacin (15 µg/mL; purity 100.50%). The reference standards against *C. albicans and A. niger* were Fluconazole (0.5 mg/mL, purity 99.83%) and Itraconazole (20 mg/mL, purity 99.50%), respectively.

##### Antimicrobial susceptibility test

The antimicrobial activity of the crude extract and fractions was evaluated using the agar well diffusion method as described by Magaldi et al*.* [19] with modifications. Twenty milliliters of Muller Hinton Agar (Oxoid, UK) for culturing *S. aureus, S. saprophyticus, S. typhi, E. coli* and *C. albicans*, and Malt Extract Agar for culturing *A. niger*, were poured into petri dishes (diameter—9 mm) and incubated at 32.5 ± 2.5 °C for 24 h and 22.5 ± 2.5 °C for 48 h, respectively to check the sterility of the plates. Aliquots of 100 µL of the inoculum were spread over the surface using a sterile spreader. Wells were bored in each plate using a cork borer (diameter—6 mm). Eighty microliters of the working concentrations of the extract/fractions (100, 50, 25, 12.5 and 6.25 mg/mL) prepared by serial dilutions, and controls were pipetted into the wells. The extracts/fractions were allowed to diffuse at room temperature (25–28 °C) for 2 h. Sterile distilled water was used as negative control and all plates were made in triplicates. Muller Hinton Agar plates were incubated at 32.5 ± 2.5 °C for 24 h while Malt Extract Agar plates were incubated at 22.5 ± 2.5 °C for 48 h and zones of growth inhibition were recorded in millimeters (mm).

##### Minimum Inhibitory Concentration (MIC)

The MIC of the crude extract and fractions were determined by the broth dilution method as described by Eloff with modification [20]. Using 96-well microtitre plates, 100 µL of sterile nutrient broth were pipetted into all the wells. Different concentrations (100 µL) of the extracts/fractions serially diluted with sterile nutrient broth were introduced into the wells and one hundred microliters of the inoculums (10.^8^ CFU/mL) were separately added to the wells containing the extract/fractions to obtain final concentrations of 50, 25, 12.5, 6.25, 3.13, 1.56, 0.78 and 0.39 mg/mL of the extracts/fractions in a total volume of 200 µL. Control experiments were also set up using sterile nutrient broth and test organism without the extract/fractions. Plates with *S. aureus, S. saprophyticus, S. typhi, E. coli* and *C. albicans* were incubated at 32.5 ± 2.5 °C for 24 h while the plate with *A. niger* was incubated at 22.5 ± 2.5 °C for 48 h. After incubation, 40 µL of 0.2 mg/mL *ρ*-iodonitrotetrazohium violet (INT) (Fluka, Germany) was added to the wells of bacterial and fungal cells, re-incubated at 32.5 ± 2.5 °C and 22.5 ± 2.5 °C, respectively for 30 min and observed for colour change. Wells with pink colour indicated viability of the organisms while wells without colour change indicated inhibition of the microorganisms. The lowest concentration that did not allow growth (no colour change) was taken as the MIC value [20, 21]

##### Minimum Lethal Concentration (MLC)

After the evaluation of the MIC, a loopful of wells without growth were seeded on sterile nutrient agar and malt extract agar plates for the MLC determination, a measure of its bactericidal (Minimum Bactericidal Concentration, MBC) and fungicidal (Minimum Fungicidal Concentration, MFC) effects. Plates of *S. aureus, S. saprophyticus, S. typhi, E. coli* and *C. albicans* were incubated at 32.5 ± 2.5 °C for 24 h whiles *A. niger* was incubated at 22.5 ± 2.5 °C for 48 h. The MLC value is the lowest concentration without visible growth on the plates [22].

### Data presentation and statistical analysis

The data were presented and analyzed using GraphPad Prism software. One-way ANOVA and Tukey's multiple comparisons test were used to analyze for statistical differences (*p* < 0.05) in the Antimicrobial activity and *p* < 0.0001 for the quantification of phytochemicals analysis.

## Results and discussion

### Phytochemical analysis of extracts

Preliminary phytochemical profiling of the hydroethanolic (70%) crude extract of *T. bangwensis* leaves and its fractions showed the presence of phenolic compounds, flavonoids, saponins, phytosterols and reducing sugars although their distribution in the fractions was polarity dependent (Supplemtary data). The phytochemicals detected are consistent with previous studies by Ekhaise et al. who also detected alkaloid, tannin, saponin, steroid and flavonoid in the methanolic extract of *T. bangwensis* leaves [11]. From the qualitative analysis of the phytochemicals in the fractions, there was as an indication of overlapping of class of compounds. Overlapping of compounds is one of the limitations in separation by solvent partitioning method. The overlapping phytochemicals was therefore quantified to evaluate their relative distribution in the fractions as shown on Table 1. From the results, the quantity of saponins, phenolic compounds and flavonoids were significantly high (*p* < 0.001) in the ethyl acetate fraction (66.47 ± 1.72% w/w, 77.75 ± 1.06 mg/100 mg GAE, 44.34 ± 0.06 mg/100 mg QE, respectively) compared to the crude, chloroform and aqueous extract /fractions. Reducing sugars showed comparable levels in all the fractions. The findings suggest that the three classes of phytochemicals (saponins, phenolic compounds and flavonoids) are mid-polar compounds, hence were well extracted into the ethyl acetate fraction than the other fractions.

**Table 1:** Quantitative analysis of the phytochemical constituents of 70% ethanolic crude extract and fractions of *T. bangwensis* leaves

Phytochemicals	Extract/Fraction				
	Crude	Pet Ether	Chloroform	Ethyl Acetate	Aqueous
Total Saponins (% w/w)	8.96 ± 2.25	-	4.08 ± 0.00	66.47 ± 1.72^*^	5.39 ± 0.29
Total phenolic compounds (mg/100 mg GAE)	9.22 ± 0.60	-	3.95 ± 0.03	77.75 ± 1.06^*^	11.89 ± 0.25
Total flavonoids mg/100 mg QE	8.76 ± 0.22	-	-	44.34 ± 0.06^*^	8.75 ± 0.21
Total reducing sugars (% w/w)	36.52 ± 0.10	32.54 ± 0.31	33.78 ± 0.66	33.37 ± 0.18	34.75 ± 0.10

The phytochemicals were quantified using established protocols. Values are Mean ± SD (*n* = 3). * represent significant difference *p* < 0.0001 relative to values of other extracts

### TLC fingerprinting

**Fig. 1:**
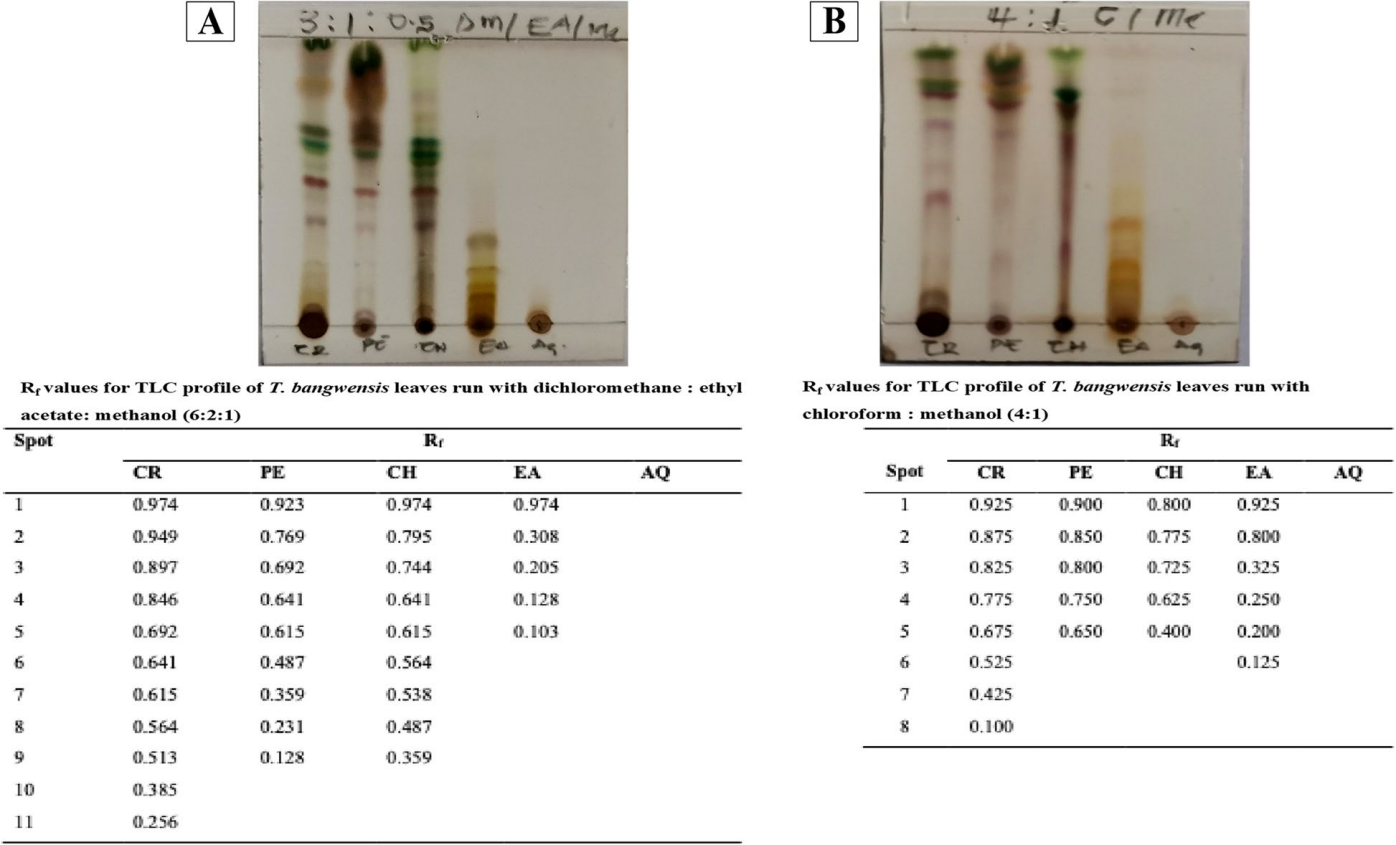
TLC profile of 70% ethanolic crude extract and fractions of *T. bangwensis* leaves with tabulated corresponding R_f_ values. The TLC plates were run with the solvent systems (**A**) dichloromethane: ethyl acetate: methanol (6:2:1) and (**B**) chloroform: methanol (4:1). CR—crude extract; PE—petroleum ether; CH- chloroform; EA—ethyl acetate; AQ—aqueous extract

The TLC profiling of the hydroethanolic (70%) crude extracts and different organic fractions of *T. bangwensis* leaves with their derived R_f_ values is shown in Fig. 1. From the TLC profiles, apart from the resultant aqueous fraction that showed no spot, all the organic fractions and the crude extract showed varying number of spots. This suggests that the phytochemicals in *T. bangwensis* leaves were from non-polar to partially polar class of phytochemicals. Using dichloromethane: ethyl acetate: methanol (6:2:1) as mobile phase, more non-polar components were observed especially in the crude, petroleum ether and chloroform fractions (Fig. 1) whereas using chloroform: methanol (4:1), more partially polar compounds were observed evidenced in the increase number of spots in the ethyl acetate fraction (Fig. 1). Both TLC profiles may be useful fingerprint for standardization of *T. bangwensis* leaves in the future.

### Antimicrobial activity

The antimicrobial activity of the hydroethanolic (70%) crude extract of *T. bangwensis* leaves and its organic fractions against the tested microorganisms (Gram-positive bacteria, Gram-negative bacteria and fungi) are shown on Table 2. From the results, the two Gram-positive bacteria tested (*S. aureus* and *S. saprophyticus*) were susceptible to both the crude extract and its fractions. *E. coli* and *S. typhi* (the two Gram-negative bacteria tested) showed susceptibility to the ethyl acetate fraction. In addition, whereas *E. coli* showed susceptibility to the petroleum ether fractions, *S. typhi* was susceptible to the chloroform fractions. All the extracts (both crude and fractions) showed inhibitory activity against the yeast, *C. albicans* but none of the extracts showed activity against the mould, *A. niger*. Taken together, Table 2 shows that the ethyl acetate fraction of *T. bangwensis* leaves exhibited strong antimicrobial activity against all the tested microorganisms, with the exception of *A. niger*, assessed by the agar-diffusion method.

**Table 2:** Antimicrobial activity of 70% ethanolic crude extract and fractions of *T. bangwensis* leaves against test organisms

Extracts	Organisms	Antimicrobial Activity (Zones of Inhibition in mm)					
		**100 mg/mL**	**50 mg/mL**	**25 mg/mL**	**12.5 mg/mL**	**6.25 mg/mL**	**Standard**
**Aqueous Fraction**	*S. aureus*	14.67 ± 0.58	13.00 ± 0.00	12.00 ± 0.00	9.67 ± 0.58	6.00 ± 0.00	23.67 ± 0.58
	*S. saprophyticus*	15.00 ± 0.00^a^	13.67 ± 0.58^b^	12.00 ± 0.00	11.00 ± 0.00	8.00 ± 0.00	25.33 ± 0.58
	*E. coli*	6.00 ± 0.00	6.00 ± 0.00	6.00 ± 0.00	6.00 ± 0.00	6.00 ± 0.00	18.67 ± 0.58
	*S. typhi*	6.00 ± 0.00	6.00 ± 0.00	6.00 ± 0.00	6.00 ± 0.00	6.00 ± 0.00	25.00 ± 0.00
	*C. albicans*	14.67 ± 0.58^c^	12.00 ± 0.00^d^	9.67 ± 0.58	6.00 ± 0.00	6.00 ± 0.00	24.67 ± 0.58
	*A. niger*	6.00 ± 0.00	6.00 ± 0.00	6.00 ± 0.00	6.00 ± 0.00	6.00 ± 0.00	21.00 ± 1.00
**Chloroform Fraction**	*S. aureus*	13.33 ± 0.58	11.00 ± 0.00	6.00 ± 0.00	6.00 ± 0.00	6.00 ± 0.00	23.67 ± 0.58
	*S. saprophyticus*	15.00 ± 0.00^a^	13.67 ± 0.58^b^	12.00 ± 0.00	11.00 ± 0.00	8.00 ± 0.00	25.33 ± 0.58
	*E. coli*	6.00 ± 0.00	6.00 ± 0.00	6.00 ± 0.00	6.00 ± 0.00	6.00 ± 0.00	18.67 ± 0.58
	*S. typhi*	11.67 ± 0.58	6.00 ± 0.00	6.00 ± 0.00	6.00 ± 0.00	6.00 ± 0.00	25.00 ± 0.00
	*C. albicans*	13.67 ± 0.58	11.00 ± 0.00	6.00 ± 0.00	6.00 ± 0.00	6.00 ± 0.00	24.67 ± 0.58
	*A. niger*	6.00 ± 0.00	6.00 ± 0.00	6.00 ± 0.00	6.00 ± 0.00	6.00 ± 0.00	21.00 ± 1.00
**Ethyl Acetate Fraction**	*S. aureus*	23.00 ± 0.00^e^	21.00 ± 0.00	20.00 ± 0.00	19.00 ± 0.00	18.00 ± 0.00	23.67 ± 0.58^e^
	*S. saprophyticus*	27.67 ± 0.58	25.33 ± 0.58^f^	22.00 ± 0.00	20.00 ± 0.00	18.00 ± 0.00	25.33 ± 0.58^f^
	*E. coli*	18.33 ± 0.58^ g^	16.00 ± 0.00	15.00 ± 0.00	14.00 ± 0.00	13.00 ± 0.00	18.67 ± 0.58^ g^
	*S. typhi*	14.00 ± 1.00	11.67 ± 0.58	6.00 ± 0.00	6.00 ± 0.00	6.00 ± 0.00	25.00 ± 0.00
	*C. albicans*	19.67 ± 0.58	17.67 ± 0.58	15.00 ± 0.00	13.00 ± 0.00^ h^	11.00 ± 0.00^i^	24.67 ± 0.58
	*A. niger*	6.00 ± 0.00	6.00 ± 0.00	6.00 ± 0.00	6.00 ± 0.00	6.00 ± 0.00	21.00 ± 1.00
**Petroleum Ether Fraction**	*S. aureus*	17.67 ± 0.58	15.00 ± 0.00^j^	13.00 ± 0.00	12.00 ± 0.00^ k^	10.00 ± 0.00	23.67 ± 0.58
	*S. saprophyticus*	12.00 ± 0.00	9.67 ± 0.58	6.00 ± 0.00	6.00 ± 0.00	6.00 ± 0.00	25.33 ± 0.58
	*E. coli*	17.67 ± 0.58^ g^	15.00 ± 0.00	12.00 ± 0.00	6.00 ± 0.00	6.00 ± 0.00	18.67 ± 0.58^ g^
	*S. typhi*	6.00 ± 0.00	6.00 ± 0.00	6.00 ± 0.00	6.00 ± 0.00	6.00 ± 0.00	25.00 ± 0.00
	*C. albicans*	13.67 ± 0.58^c^	12.00 ± 0.00^d^	6.00 ± 0.00	6.00 ± 0.00	6.00 ± 0.00	24.67 ± 0.58
	*A. niger*	6.00 ± 0.00	6.00 ± 0.00	6.00 ± 0.00	6.00 ± 0.00	6.00 ± 0.00	21.00 ± 1.00
**70% Ethanol Crude Extract**	*S. aureus*	16.00 ± 0.00	15.00 ± 0.00^j^	14.00 ± 0.00	11.33 ± 0.58^ k^	7.67 ± 0.58	23.67 ± 0.58
	*S. saprophyticus*	18.67 ± 0.58	16.67 ± 0.58	14.33 ± 0.58	11.67 ± 0.58	10.00 ± 0.00	25.33 ± 0.58
	*E. coli*	6.00 ± 0.00	6.00 ± 0.00	6.00 ± 0.00	6.00 ± 0.00	6.00 ± 0.00	18.67 ± 0.58
	*S. typhi*	6.00 ± 0.00	6.00 ± 0.00	6.00 ± 0.00	6.00 ± 0.00	6.00 ± 0.00	25.00 ± 0.00
	*C. albicans*	16.00 ± 0.00	15.00 ± 0.00	14.00 ± 0.00	12.33 ± 0.58^ h^	10.33 ± 0.58^i^	24.67 ± 0.58
	*A. niger*	6.00 ± 0.00	6.00 ± 0.00	6.00 ± 0.00	6.00 ± 0.00	6.00 ± 0.00	21.00 ± 1.00

The susceptibility of the microorganisms to the extracts was tested using the agar well diffusion method. Values are mean ± SD (*n* = 3). 6.00 ± 0.00 mm = No zone of inhibition

^a^^−^^k^Mean values with same alphabet represent insignificant difference (*p* > 0.05) in antimicrobial activity analyzed by Tukey’s multiple comparisons test

**Fig. 2:**
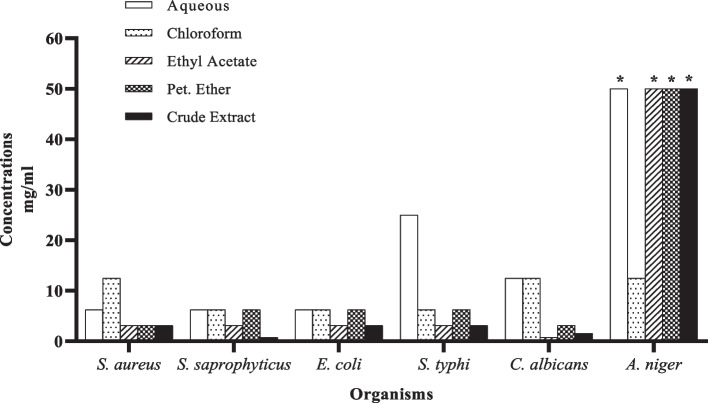
Minimum inhibitory concentrations of 70% ethanolic crude extract and fractions of *T. bangwensis* leaves against test organisms. The susceptibility of the microorganisms to the extracts was tested using the broth diffusion method. * = MIC more than 50 mg/ml

Also, using the broth dilution method, the susceptibility of the tested bacteria and yeast to the crude extracts of *T. bangwensis* leaves and its fractions was evaluated (Fig. 2). From the results, all the microorganisms tested exhibited varying degrees of susceptibility to the extracts in the order; crude extract > ethyl acetate > petroleum ether > chloroform > aqueous fraction. All the test organisms (excluding *A. niger*) were susceptible at concentrations not more than 25 mg/mL (Fig. 2). *S. saprophyticus* and *C. albicans* were more susceptible to the crude extract and ethyl acetate fraction, respectively, with MIC of 0.78 mg/mL each. *Candida albicans* was also susceptible to the crude extract with MIC of 1.56 mg/mL. Furthermore, the crude extract and ethyl acetate fraction showed strong inhibitory activity against all the other Gram-positive and Gram-negative bacteria with MIC value of 3.13 mg/mL (Fig. 2)*.* Similar to the crude extract and ethyl acetate fraction, the petroleum ether fraction also showed strong inhibitory activity against both Gram-positive and Gram-negative bacteria as well as *C. albicans* with MIC values ranging from 3.13 mg/mL—6.25 mg/mL. The chloroform fraction and resulting aqueous fraction after partitioning showed relatively lower inhibitory activity with MIC within the range of 6.25 mg/mL—25 mg/mL. Notwithstanding the apparent resistance of *A. niger* to all the extracts, the chloroform fraction exhibited activity against both fungi tested (*A. niger* and *C. albicans*) with MIC of 12.5 mg/mL.

From the study there appeared to be some inconsistency between the susceptibility tests by the agar well diffusion method and the broth dilution method. For instance, using the agar well diffusion method it was observed that *E. coli* and/or *S. tphyi* were not susceptible to some of the extracts which proved false in the broth dilution method. This could be attributed to the limitation in the use of agar diffusion method for plant extracts, where the difference in the polarity of the constituents of the plant extracts leads to different rates of diffusion in the agar (an aqueous preparation), thus affecting the zones of inhibitions [23, 24]. This however, is not observed with the broth dilution method. Notwithstanding, the agar well diffusion was useful in this experiment to give useful preliminary data on the antimicrobial properties of the crude extract of *T. bangwensis* and its fractions. Therefore, taken together (results from the agar well diffusion and broth dilution methods), it can be deduced that the Gram-positive bacteria were the most susceptible organisms to the extracts, Gram-negative bacteria showed intermediate susceptibility while the fungi showed the least susceptibility. These findings support a study by Efuntoye et al*.* and Ekhaise et al. which demonstrated that *T. bangwensis* (although harvested from a different geographical location and different host plant) possesses antimicrobial activity against both Gram-positive and Gram- negative bacteria [8, 11].

**Fig. 3:**
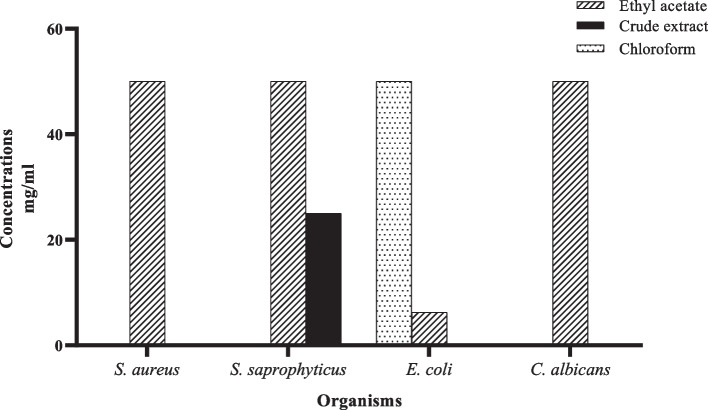
Minimum lethal concentrations of 70 % ethanolic crude extract and fractions of *T. bangwensis* leaves against test organisms. The susceptibility of the microorganisms to the extracts was tested using the broth diffusion method

The study also evaluated the bactericidal and fungicidal effects of the extracts on the tested microorganisms. The results showed that crude extract of *T. bangwensis* leaves, its ethyl acetate and chloroform fractions exhibited lethal microbial activity (Fig. 3). The MLC of the crude extract and its fractions on the tested organisms was in the range of 6.25 mg/mL to 50 mg/mL. The ethyl acetate fraction was lethal to *E. coli* at MBC of 6.25 mg/mL, and to *S. aureus* and *S. saprophyticus* at MBC of 50 mg/mL each. It also showed fungicidal activity against *C. albicans* with MFC of 50 mg/mL. The crude extract was lethal to only *S. saprophyticus* at MBC of 25 mg/mL while the chloroform fraction was lethal to *E. coli* at MBC 50 mg/mL (Fig. 3). However, the observed MLC values were higher than the MLC values of other medicinal plants reported in literature [25–28].The authors did not come across any report on the bactericidal and fungicidal properties of *T. bangwensis* in literature. Notwithstanding, the MIC values of *T. bangwensis* and its fractions, which complements the MLC values were very comparable to what was reported by Ekhaise et al.(10 – 50 mg/mL). Thus, the findings highlight the susceptibility of *E. coli, S. aureus*, *S. saprophyticus and C. albicans* to *T. bangwensis* and its fractions.

The antimicrobial activities reported in this study can be associated with the various phytochemical constituents detected in the crude extract of *T. bangwensis* leaves and its fractions. Phytochemical compounds such as alkaloids, saponins, tannins, flavonoids and steroids have been known to be biologically active and thus may be responsible for the antimicrobial activities of plants, hence their use in traditional medicine [29]. From the study, the ethyl acetate showed higher levels of the quantified phytochemicals (saponins, phenolic compounds and flavonoids) compared to the petroleum ether, chloroform and aqueous fractions. Therefore, the high levels of these phytochemicals probably underpin the relatively stronger antimicrobial activity of the ethyl acetate fraction; exhibiting lethal microbial activity against *E. coli*, *S. aureus*, *S. saprophyticus* and *C. albicans.* The phytochemicals in the other fractions could also be responsible for the various observed antimicrobial activities. However, the effect of overlapping of compounds resulting from the solvent partitioning method could also account for some of the comparable antimicrobial activities observed. Therefore, having obtained preliminary data on the antimicrobial properties of the phytochemicals in the various organic factions of *T. bangwensis* leaves, bioactive compounds would be isolated and characterized in future studies.

## Conclusion

The study shows that the phytochemical composition of the crude extract of *Tapinanthus bangwensis* leaves, mostly extracted into ethyl acetate, include phenolic compounds, flavonoids, saponins, phytosterols and reducing sugars. Also, the results showed that *E. coli*, *S. aureus*, *S. saprophyticus* and *C. albicans* are susceptible to the crude extract of *T. bangwensis* leaves and its fractions, thereby representing a potential source of natural antimicrobial agents. Further study is required to isolate antimicrobial compounds from the plant and evaluate the potential effectiveness against microorganisms, aimed at potential discovery and development of natural bioactive antimicrobial agents.

## Supplementary Information

**Additional file 1: Table S1.** Preliminaryqualitative analysis of the phytochemical constituents of 70 % ethanolic crudeextract of *T. bangwensis* leaves andits fractions. **Table S2.** Quantitativeanalysis of the total saponins of 70 % ethanolic crude extract and fractions of*T. bangwensis* leaves. **TableS3.** Quantitative analysis of the total phenolic compounds of 70 % ethanoliccrude extract and fractions of *T. bangwensis*leaves. **TableS4.** Quantitative analysis of the total flavonoids of 70 % ethanolic crudeextract and fractions of *T. bangwensis*leaves. **TableS5.** Quantitative analysis of the total reducing sugars of 70 % ethanolic crudeextract and fractions of *T. bangwensis*leaves. **Figure S1.** Antimicrobialactivity of 70 % ethanolic crude extract and fractions of *T. bangwensis *leaves againsttest organisms. **Table S6. **Minimum inhibitoryconcentrations of 70 % ethanolic crude extract and fractions of *T. bangwensis *leavesagainst test organisms. **Table S7. **Minimum lethal concentrationsof 70 % ethanolic crude extract and fractions of *T. bangwensis *leaves against test organisms. 

## Data Availability

Most of the relevant data is made available in this article. All others are provided as supplementary data attached to the submission.
