# Supplementary material for: Assessment of the phytochemical composition and antimicrobial properties of Tapinanthus bangwensis leaves hosted by the branches of Persea americana
Source: BMC Complement Med Ther. 2023 Feb 3;23:34. doi: 10.1186/s12906-023-03860-w (PMC9896793; doi:10.1186/s12906-023-03860-w)
Supplement: Supplementary file 1 — Additional file 1: Table S1. Preliminaryqualitative analysis of the phytochemical constituents of 70 % ethanolic crudeextract of T. bangwensis leaves andits fractions. Table S2. Quantitativeanalysis of the total saponins of 70 % ethanolic crude extract and fractions ofT. bangwensis leaves. TableS3. Quantitative analysis of the total phenolic compounds of 70 % ethanoliccrude extract and fractions of T. bangwensisleaves. TableS4. Quantitative analysis of the total flavonoids of 70 % ethanolic crudeextract and fractions of T. bangwensisleaves. TableS5. Quantitative analysis of the total reducing sugars of 70 % ethanolic crudeextract and fractions of T. bangwensisleaves. Figure S1. Antimicrobialactivity of 70 % ethanolic crude extract and fractions of T. bangwensis leaves againsttest organisms. Table S6. Minimum inhibitoryconcentrations of 70 % ethanolic crude extract and fractions of T. bangwensis leavesagainst test organisms. Table S7. Minimum lethal concentrationsof 70 % ethanolic crude extract and fractions of T. bangwensis leaves against test organisms. [file 12906_2023_3860_MOESM1_ESM.docx]

**Supplementary Data**

**Table S1: Preliminary qualitative analysis of the phytochemical constituents of 70 % ethanolic crude extract of *T. bangwensis* leaves and its fractions**

| **Phytochemical constituents** | *T. bangwensis* (70% crude extract) | *T. bangwensis* (Pet. Ether fraction*)* | *T.*  *bangwensis* (Chloroform fraction) | *T. bangwensis (*Ethyl acetate fraction) | *T. bangwensis* (Aqueous fraction) |
| --- | --- | --- | --- | --- | --- |
| Phenolic compounds | **+** | **-** | **+** | **+** | **+** |
| Flavonoids | **+** | **-** | **-** | **+** | **+** |
| Saponins | **+** | **-** | **+** | **+** | **+** |
| Phytosterols | **+** | **+** | **+** | **-** | **-** |
| Reducing sugar | **+** | **+** | **+** | **+** | **+** |

**+** Present - Absent

**Table S2: Quantitative analysis of the total saponins of 70 % ethanolic crude extract and fractions of *T. bangwensis* leaves**

| Extract | Total saponins (% w/w) | | |
| --- | --- | --- | --- |
|  | 1 | 2 | 3 |
| \| Crude \| \| --- \| | 11.22 | 6.71 | 8.96 |
| Petroleum ether | ND | ND | ND |
| Chloroform | 4.077 | 4.076 | 4.08 |
| Ethyl acetate | 68.18 | 64.75 | 66.47 |
| Aqueous | 5.68 | 5.11 | 5.39 |

ND – not detected from the qualitative phytochemical analysis

**Table S3: Quantitative analysis of the total phenolic compounds of 70 % ethanolic crude extract and fractions of *T. bangwensis* leaves**

| Extract | Total phenolic compounds (mg/100 mg GAE) | | |
| --- | --- | --- | --- |
|  | 1 | 2 | 3 |
| \| Crude \| \| --- \| | 8.217 | 610.285 | 9.169 |
| Petroleum ether | ND | ND | ND |
| Chloroform | 3.890 | 3.999 | 3.972 |
| Ethyl acetate | 77.768 | 77.006 | 78.473 |
| Aqueous | 11.400 | 12.108 | 12.162 |

ND – not detected from the qualitative phytochemical analysis

**Table S4: Quantitative analysis of the total flavonoids of 70 % ethanolic crude extract and fractions of *T. bangwensis* leaves**

| Extract | Total flavonoids (mg/100 mg QE) | | |
| --- | --- | --- | --- |
|  | 1 | 2 | 3 |
| \| Crude \| \| --- \| | 9.235 | 8.900 | 9.198 |
| Petroleum ether | ND | ND | ND |
| Chloroform | ND | ND | ND |
| Ethyl acetate | 44.823 | 44.971 | 43.240 |
| Aqueous | 8.342 | 9.012 | 8.900 |

ND – not detected from the qualitative phytochemical analysis

**Table S5: Quantitative analysis of the total reducing sugars of 70 % ethanolic crude extract and fractions of *T. bangwensis* leaves**

| Extract | Total reducing sugars (% w/w) | | |
| --- | --- | --- | --- |
|  | 1 | 2 | 3 |
| \| Crude \| \| --- \| | 36.5217094 | 36.6965532 | 36.3468656 |
| Petroleum ether | 32.9082708 | 32.7917082 | 31.9174892 |
| Chloroform | 33.607646 | 35.0063964 | 32.733427 |
| Ethyl acetate | 33.2579584 | 33.7242085 | 33.1413959 |
| Aqueous | 34.9481152 | 34.5984276 | 34.7149901 |

ND – not detected from the qualitative phytochemical analysis

**Figure S1: Antimicrobial activity of 70 % ethanolic crude extract and fractions of *T. bangwensis* leaves against test organisms**

The susceptibility of the microorganisms to the extracts was tested using the agar well diffusion method.

**Table S6 :** Minimum inhibitory concentrations of 70 % ethanolic crude extract and fractions of *T. bangwensis* leaves against test organisms.

| **Organisms** | Minimum inhibitory concentrations (mg/mL) | | | | |
| --- | --- | --- | --- | --- | --- |
|  | **Aqueous Fraction** | **Chloroform Fraction** | **Ethyl Acetate Fraction** | **Petroleum Ether Fraction** | **70% Ethanol Crude Extract** |
| *S. aureus* | 6.25 | 12.5 | 3.13 | 3.13 | 3.13 |
| *S. saprophyticus* | 6.25 | 6.25 | 3.13 | 6.25 | 0.78 |
| *E. coli* | 6.25 | 6.25 | 3.13 | 6.25 | 3.13 |
| *S. typhi* | 25 | 6.25 | 3.13 | 6.25 | 3.13 |
| *C. albicans* | 12.5 | 12.5 | 0.78 | 3.13 | 1.56 |
| *A. niger* | >50 | 12.5 | >50 | >50 | >50 |

The susceptibility of the microorganisms to the extracts was tested using the broth diffusion method

**Table S7:** Minimum lethal concentrations of 70 % ethanolic crude extract and fractions of *T. bangwensis* leaves against test organisms

| **Organisms** | Minimum lethal concentrations (mg/mL) | | | | |
| --- | --- | --- | --- | --- | --- |
|  | **Aqueous Fraction** | **Chloroform Fraction** | **Ethyl Acetate Fraction** | **Petroleum Ether Fraction** | **70% Ethanol Crude Extract** |
| *S. aureus* | >50 | >50 | 50 | >50 | >50 |
| *S. saprophyticus* | >50 | >50 | 50 | >50 | 25 |
| *E. coli* | >50 | >50 | 6.25 | >50 | >50 |
| *S. typhi* | >50 | >50 | >50 | >50 | >50 |
| *C. albicans* | >50 | >50 | 50 | >50 | >50 |
| *A. niger* | >50 | >50 | >50 | >50 | >50 |

The susceptibility of the microorganisms to the extracts was tested using the broth diffusion method
